# Sphingolipid Profiling Reveals Different Extent of Ceramide Accumulation in Bovine Retroperitoneal and Subcutaneous Adipose Tissues

**DOI:** 10.3390/metabo10110473

**Published:** 2020-11-19

**Authors:** Yue Hei Leung, Sonja Christiane Bäßler, Christian Koch, Theresa Scheu, Ulrich Meyer, Sven Dänicke, Korinna Huber, Ákos Kenéz

**Affiliations:** 1 Department of Infectious Diseases and Public Health, City University of Hong Kong, Hong Kong; yuehleung4-c@my.cityu.edu.hk; 2 Institute of Animal Science, University of Hohenheim, 70599 Stuttgart, Germany; Sonja_10394@gmx.de (S.C.B.); Korinna.Huber@uni-hohenheim.de (K.H.); 3 Educational and Research Centre for Animal Husbandry, Hofgut Neumuehle, 67728 Muenchweiler a.d. Alsenz, Germany; c.koch@neumuehle.bv-pfalz.de (C.K.); T.Scheu@neumuehle.BV-Pfalz.de (T.S.); 4 Institute of Animal Nutrition, Friedrich-Loeffler-Institute (FLI), Federal Research Institute for Animal Health, 38116 Braunschweig, Germany; ulrich.meyer@fli.de (U.M.); sven.daenicke@fli.de (S.D.)

**Keywords:** sphingolipids, ceramides, adipose tissue biology, retroperitoneal adipose tissue, subcutaneous adipose tissue, bovine

## Abstract

Sphingolipids are bioactive lipids that can modulate insulin sensitivity, cellular differentiation, and apoptosis in a tissue-specific manner. However, their comparative profiles in bovine retroperitoneal (RPAT) and subcutaneous adipose tissue (SCAT) are currently unknown. We aimed to characterize the sphingolipid profiles using a targeted lipidomics approach and to assess whether potentially related sphingolipid pathways are different between SCAT and RPAT. Holstein bulls (*n* = 6) were slaughtered, and SCAT and RPAT samples were collected for sphingolipid profiling. A total of 70 sphingolipid species were detected and quantified by UPLC-MS/MS in multiple reaction monitoring (MRM) mode, including ceramide (Cer), dihydroceramide (DHCer), sphingomyelin (SM), dihydrosphingomyelin (DHSM), ceramide-1-phosphate (C1P), sphingosine-1-phosphate (S1P), galactosylceramide (GalCer), glucosylceramide (GluCer), lactosylceramide (LacCer), sphinganine (DHSph), and sphingosine (Sph). Our results showed that sphingolipids of the de novo synthesis pathway, such as DHSph, DHCer, and Cer, were more concentrated in RPAT than in SCAT. Sphingolipids of the salvage pathway and the sphingomyelinase pathway, such as Sph, S1P, C1P, glycosphingolipid, and SM, were more concentrated in SCAT. Our results indicate that RPAT had a greater extent of ceramide accumulation, thereby increasing the concentration of further sphingolipid intermediates in the de novo synthesis pathway. This distinctive sphingolipid distribution pattern in RPAT and SCAT can potentially explain the tissue-specific activity in insulin sensitivity, proinflammation, and oxidative stress in RPAT and SCAT.

## 1. Introduction

**Figure 1:**
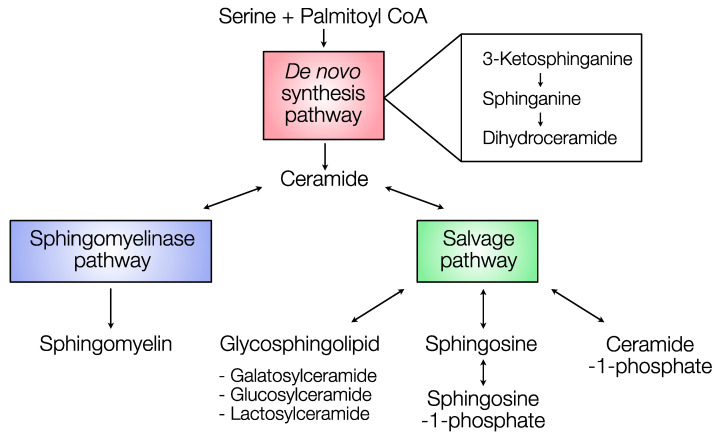
Sphingolipid metabolic pathways according to Merrill [8], and Maceyka and Spiegel [9]. The de novo synthesis pathway (red) begins with the condensation of serine and palmitoyl coenzyme A (CoA) to form ceramide through a series of reactions including the conversion of 3-ketosphinganine, sphinganine, and dihydroceramide. Ceramide can be modified into sphingomyelin via the sphingomyelinase pathway (blue) or into glycosphingolipid, sphingosine, and ceramide-1-phosphate via the salvage pathway (green). Double arrows indicate reversible reactions.

Sphingolipids are a class of structural lipids in eukaryotic cells that not only constitute the cell membrane but also exhibit a cell signaling function to modulate insulin sensitivity, differentiation, and apoptosis in a tissue-specific manner [1,2]. Although associations between bovine metabolic health and sphingolipid function have partially been established [3], most of this research merely focused on ceramides. Even fewer studies have discussed the physiological role of sphingolipids in bovine adipose tissue. To better understand the sphingolipid function in bovine adipose tissue, it is important to consider the dynamics in the metabolic pathways of sphingolipid synthesis, degradation, and modification: the de novo synthesis pathway, the salvage pathway, and the sphingomyelinase pathway (Figure 1). The de novo synthesis pathway is essential to the survival and normal metabolic activity of adipocytes. It was demonstrated that an interruption of the de novo synthesis pathway by adipocyte-specific serine palmitoyltransferase (SPT) mutation reduces adipose tissue size, decreases the downstream sphingolipid quantity, and significantly decreases the circulating level of adipokines leptin and adiponectin in mice [4]. The salvage pathway is important for sphingolipid recycling and turnover. Large sphingolipids such as glycosphingolipids and ceramide-1-phosphates (C1P) are broken down into sphingosine and further transformed into ceramide [5]. The sphingomyelinase pathway or sphingomyelin hydrolysis pathway is essential for cellular function and health. It was observed that human adipose tissues affected by inflammation featured greater gene expression levels of sphingomyelinase [6]. This implied that sphingomyelin-driven ceramides could be associated with inflammation under the transformation by sphingomyelinase [7]. Studying sphingolipid biology, particularly in adipose tissue, is critical in dairy cattle because the physiological functions of sphingolipids are suggested to represent a potential link between metabolic stress and physiological adaptation [2].

Adipose tissue metabolism is important for sphingolipid biology not only because of its role in lipid storage and release, but also because of its active role in regulating homeostasis and inflammatory response [10]. However, adipose metabolism differs to some extent between adipose depots. Retroperitoneal (RPAT) and subcutaneous adipose tissue (SCAT) in dairy cows are suggested to be different in insulin signaling, proinflammatory signaling, and lipolytic activity [11,12,13]. It was demonstrated that RPAT is more responsive than SCAT regarding the insulin signaling pathway, with greater protein kinase B (Akt) and adenosine monophosphate-activated protein kinase (AMPK) phosphorylation, as well as greater fatty acid synthase (FAS) expression, shown in an ex vivo study [11]. Moreover, RPAT had greater proinflammatory cytokine and chemokine messenger RNA (mRNA) expression than SCAT during energy overfeeding [12]. Locher et al. demonstrated that the hormone-sensitive lipase (HSL) activity was higher in RPAT than SCAT as reflected by a greater extent of phosphorylation at Ser 660, an activation marker of HSL [13]. Additionally, RPAT had higher lipolytic activity than SCAT under catecholamine stimulation [14]. In contrast to SCAT, RPAT accumulated and lost adipose mass with greater fluctuation during the peripartum period [15]. Collectively, these studies demonstrated that RPAT responds more dynamically to metabolic stimuli than SCAT, which is in agreement with human adipose biology [16].

To study the systemic relationship of sphingolipids in adipose tissues, particularly in SCAT and RPAT, the comparative distribution pattern of various sphingolipid moieties in these adipose depots has to be determined. However, the sphingolipid profiles of bovine SCAT and RPAT have not yet been reported. We hypothesized that the sphingolipid profiles differ between RPAT and SCAT, where particularly ceramides may be more concentrated in RPAT than in SCAT, given that RPAT was previously shown to be more prone to proinflammatory signaling than SCAT. In this study, we aimed to characterize the sphingolipid profiles using a lipidomics approach and to determine whether SCAT and RPAT differ in the concentration of various sphingolipid species. Hence, the objectives of this study were to compare and contrast the sphingolipid species in SCAT and RPAT and to distinguish the major differences in the sphingolipid biochemical pathways of both tissues.

## 2. Results

### 2.1. Comparative Sphingolipid Distribution in SCAT and RPAT

**Figure 2:**
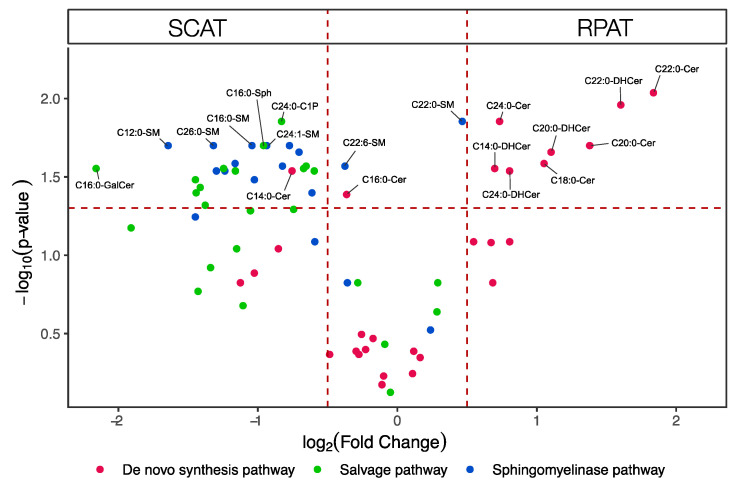
Volcano plot visualizing the fold change (retroperitoneal (RPAT) to subcutaneous adipose tissue (SCAT)) over the *p*-value of sphingolipids from six pairs of adipose tissues. The *p*-value of each sphingolipid species was calculated by paired Student’s *t*-test with false discovery rate (FDR) correction. The dotted horizontal line indicates a statistical threshold of *p* < 0.05, and the dotted vertical lines between −0.5 and 0.5 log_2_(fold change) indicate the 1-fold restriction threshold. The figure shows that sphingolipid species in the de novo synthesis pathway were more concentrated in RPAT. In contrast, sphingolipid species in the salvage pathway and the sphingomyelinase pathway were more concentrated in SCAT. Cer: ceramide; C1P: ceramide-1-phosphate; DHCer: dihydroceramide; GalCer: galactosylceramide; Sph: sphingosine; SM: sphingomyelin.

**Figure 3:**
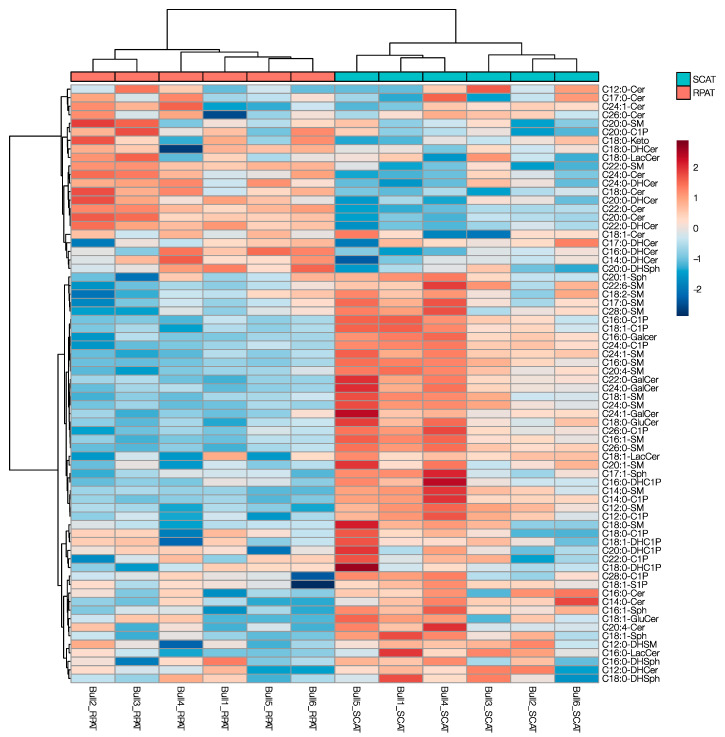
The heatmap with dendrograms visualizing the individual variability between the concentration of various sphingolipids in SCAT and RPAT of Holstein bulls. Data were log-transformed and Pareto-scaled. Samples (bulls) and features (metabolites) were clustered in Euclidean distance with the Ward algorithm.

The fold-changes (FCs) of RPAT to SCAT over respective *p*-values of sphingolipids are shown in the volcano plot in Figure 2. In the de novo synthesis pathway (red), eight out of 28 sphingolipid species were more concentrated in RPAT (*p* < 0.1, log_2_FC > 0.5), while two species of sphingolipids in this category that were more concentrated in SCAT were C14:0-Cer (*p* = 0.029, log_2_FC = −0.76) and C16:0-Cer (*p* = 0.041, log_2_FC = −0.36). In the salvage pathway (green), 12 out of 24 sphingolipid species were more concentrated in SCAT (*p* < 0.1, log_2_FC < −0.5), while none of them were more concentrated in RPAT with a significant difference. In the sphingomyelinase pathway (blue), 12 out of 18 sphingolipid species were more concentrated in SCAT (*p* < 0.1, log_2_FC < 0.5), and C22:0-sphingomyelin (SM) was the only sphingolipid in this category which was more concentrated in RPAT, reaching the fold change threshold (*p* = 0.014, log_2_FC = 0.47). Collectively, RPAT was more concentrated with the sphingolipids of the de novo synthesis pathway, and SCAT was more concentrated with the sphingolipids of the salvage and sphingomyelinase pathways. This separation between SCAT and RPAT sphingolipid profiles was in line with the separation pattern of the tissues and sphingolipids shown in the principal component analysis (PCA) biplots (Figure S1, Supplementary Materials). Furthermore, the hierarchical clustering showed that the adipose tissues clustered in the first clades of the dendrogram, indicating that the separation according to adipose depot had a bigger impact on the overall sphingolipid profile than the variation between individual animals (Figure 3).

### 2.2. Distribution of Sphingolipids in the De Novo Synthesis Pathway

**Figure 4:**
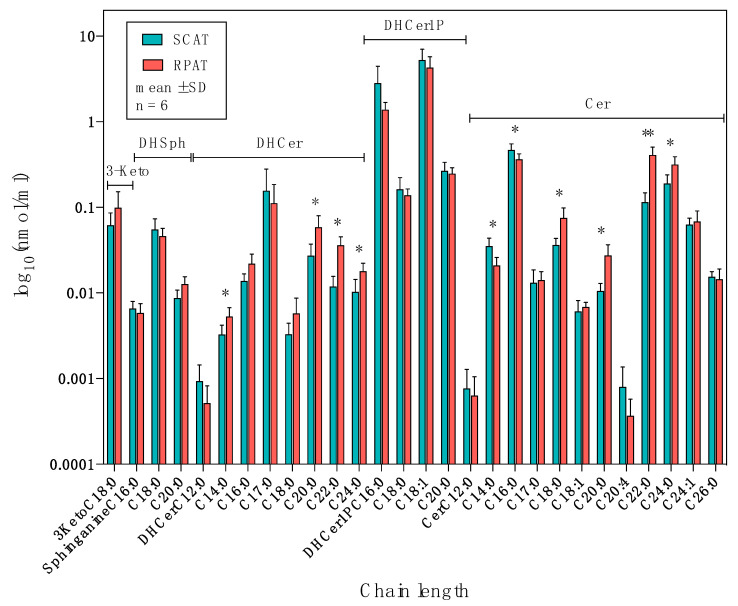
The distribution of sphingolipids in the de novo synthesis pathway in subcutaneous (SCAT) and retroperitoneal adipose tissue (RPAT) in German Holstein bulls (*n* = 6). Data are presented as the mean ± standard deviation (SD) on a logarithmic scale. 3-Keto: 3-ketosphinganine concentration; DHSph: sphinganine concentration; DHCer: dihydroceramide concentration; DHCer1P: dihydroceramide-1-phosphate concentration; Cer: ceramide concentration. Asterisks indicate significant differences (* *p* < 0.05; ** *p* < 0.01) between two adipose tissues by paired Student’s *t*-test with FDR correction.

The distribution of sphingolipids of the de novo synthesis pathway in SCAT and RPAT is shown in Figure 4. The concentrations of 3-ketosphinganine and sphinganines were not significantly different between SCAT and RPAT. Among the dihydroceramides, C17:0-DHCer did not show significant differences, while C12:0-DHCer was more concentrated in SCAT than RPAT (*p* = 0.091, log_2_FC = −0.85), and the remaining DHCers were more concentrated in RPAT than in SCAT: C16:0- and C18:0-DHCer (*p* < 0.1, log_2_FC > 0.5); C14:0-, C20:0-, C22:0-, and C24:0-DHCer (*p* < 0.05, log_2_FC > 0.5). Among the dihydroceramide-1-phosphates, none of the detected species, C16:0-, C18:0-, C18:1-, and C20:0-DHCer1P, showed significant differences between SCAT and RPAT. Among the ceramides, C12:0-, C17:0-, C18:1-, C20:4-, C24:1-, and C26:0-Cer did not show significant differences, while two species of Cer were more concentrated in SCAT than in RPAT: C14:0-Cer (*p* = 0.029, log_2_FC = −0.76) and C16:0-Cer (*p* = 0.041, log_2_FC = −0.36); the remaining Cers were more concentrated in RPAT than in SCAT: C18:0-, C20:0-, and C24:0-Cer (*p* < 0.05, log_2_FC > 0.5); C22:0-Cer (*p* = 0.0092, log_2_FC = 1.84). In summary, the majority of the sphingolipid species in the de novo synthesis pathway were more concentrated in RPAT, compared with SCAT, except for C14:0- and C16:0-Cer, which were more concentrated in SCAT.

### 2.3. Distribution of Sphingolipids in the Salvage Pathway

**Figure 5:**
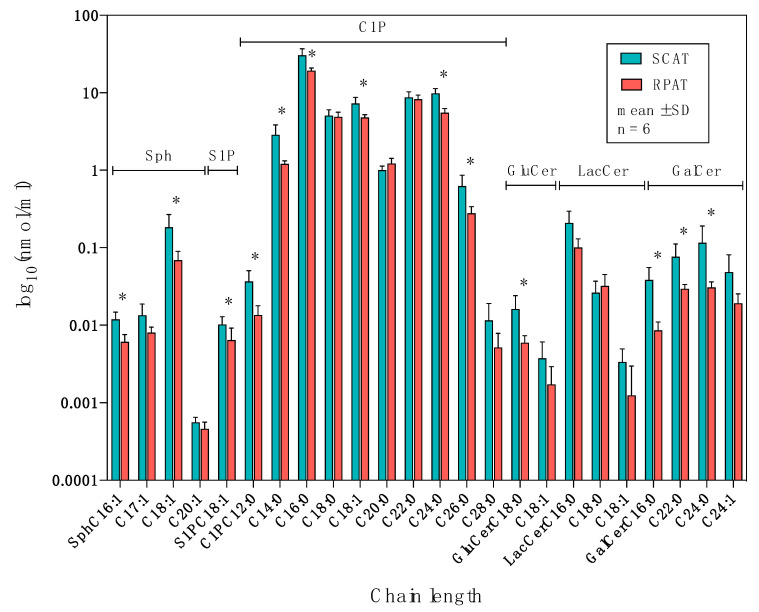
The distribution of sphingolipids in the salvage pathway in subcutaneous (SCAT) and retroperitoneal adipose tissue (RPAT) in German Holstein bulls (*n* = 6). Data are presented as the mean ± standard deviation (SD) on a logarithmic scale. Sph: sphingosine concentration; S1P: sphingosine-1-phosphate concentration; C1P: ceramide-1-phosphate concentration; GluCer: glucosylceramide concentration; LacCer: lactosylceramide concentration; GalCer: galactosylceramide concentration. Asterisks indicate significant differences (* *p* < 0.05) between two adipose tissues by paired Student’s *t*-test with FDR correction.

The distribution of sphingolipids of the salvage pathway in SCAT and RPAT is shown in Figure 5. Among the sphingosines, C20:1-Sph did not show significant differences, while the remaining Sphs were more concentrated in SCAT than in RPAT: C17:1-Sph (*p* = 0.051, log_2_FC = −0.74); C16:1- and C18:1-Sph (*p* < 0.05, log_2_FC < −0.5). Concerning sphingosine-1-phosphate, C18:1-S1P was more concentrated in SCAT than in RPAT (*p* = 0.028, log_2_FC = −0.67). Among the ceramide-1-phosphates, C18:0-, C20:0-, C22:0, and C28:0-C1P did not show significant differences, while the remaining C1Ps were more concentrated in SCAT than in RPAT: C12:0-, C14:0-, C16:0-, C18:1-, C24:0-, and C26:0-C1P (*p* < 0.05, log_2_FC < −0.5). Among the glucosylceramides, C18:1-GluCer did not show significant differences, while C18:0-GluCer was more concentrated in SCAT than in RPAT (*p* = 0.04, log_2_FC = −1.44). The concentrations of lactosylceramides were not significantly different between SCAT and RPAT. Among the galactosylceramides, C24:1-GalCer was not significantly different, while the remaining GalCers were more concentrated in SCAT than in RPAT: C16:0-, C22:0-, and C24:0-GalCer (*p* < 0.05, log_2_FC < −0.05). Collectively, all sphingolipid species in the salvage pathway were more concentrated in SCAT compared with RPAT.

### 2.4. Distribution of Sphingolipids in the Sphingomyelinase Pathway

**Figure 6:**
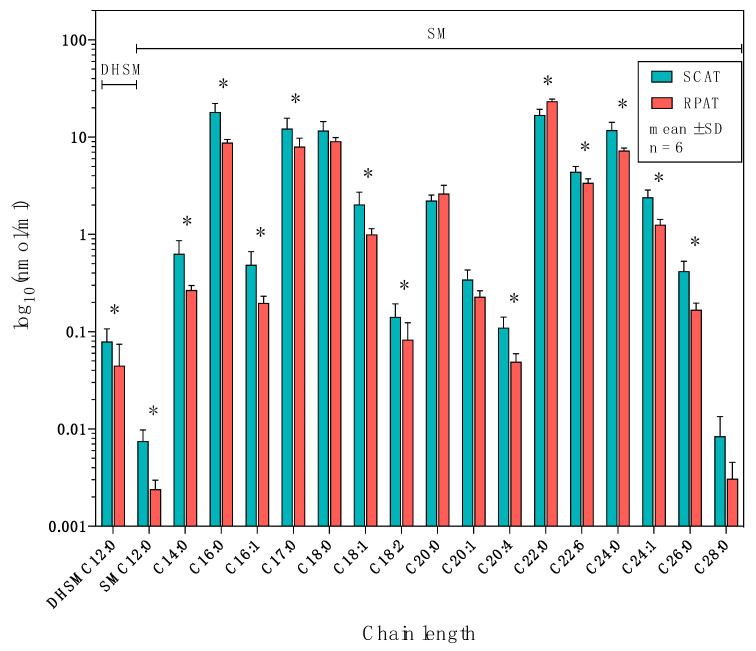
The distribution of dihydroceramide and sphingolipids of the sphingomyelinase pathway in subcutaneous (SCAT) and retroperitoneal adipose tissue (RPAT) in German Holstein bulls (*n* = 6). Data are presented as the mean ± standard deviation (SD) on a logarithmic scale. DHSM: dihydrosphingomyelin concentration; SM: sphingomyelin concentration. Asterisks indicate significant differences (* *p* < 0.05) between two adipose tissues by paired Student’s *t*-test with FDR correction.

The distributions of sphingolipids of the sphingomyelinase pathway in SCAT and RPAT are shown in Figure 6. Concerning dihydrosphingomyelin, C12:0-DHSM was more concentrated in SCAT than in RPAT (*p* = 0.027, log_2_FC = −0.82). Among the sphingomyelins, C18:0-, C20:0-, C20:1-, and C28:0-SM did not show significant differences, C22:0-SM was more concentrated in RPAT than in SCAT (*p* = 0.014, log_2_FC = 0.47), and the remaining SMs were more concentrated in SCAT than in RPAT: C12:0-, C14:0-, C16:0-, C16:1-, C17:0-, C18:1-, C18:2-, C20:4-, C24:0-, C24:1-, and C26:0-SM (*p* < 0.05, log_2_FC < −0.5); C22:6-SM (*p* = 0.027, log_2_FC < −0.38). Collectively, sphingolipids in the sphingomyelinase pathway were generally more concentrated in SCAT, compared with RPAT, except for C22:0-SM, which was more concentrated in RPAT.

## 3. Discussion

This study analyzed 70 sphingolipid species, and the concentrations of 36 species were found to be statistically different between RPAT and SCAT (*p* < 0.05), indicating that the sphingolipid profiles of these two adipose tissue depots were remarkably different. The differential concentration of sphingolipid species can reflect dissimilar sphingolipid pathway activities, affecting downstream signaling function [1]. Limitations of this study include the relatively small sample size, restricting the assessment of any possible dietary influence. Sphingolipid metabolism was shown to be altered by dietary factors in dairy cows [17]; however, whether the diet has an effect on adipose dependent distribution of sphingolipids should be tested in further studies. The other potential limitation is that our data only reflect the differential origin of two adipose tissues; however, the sphingolipid profile may be influenced by the different cellular composition in SCAT and RPAT. White adipose tissue contains several types of cells, including mesenchymal stem cells, macrophages, B and T cells, and preadipocytes, in addition to mature adipocytes [18]. The novelty of this study is confirming that bovine SCAT and RPAT are distinct in their metabolism, by providing the sphingolipid profiles of these two depots in a lipidomics approach.

### 3.1. Higher Concentration of Ceramides in RPAT

**Figure 7:**
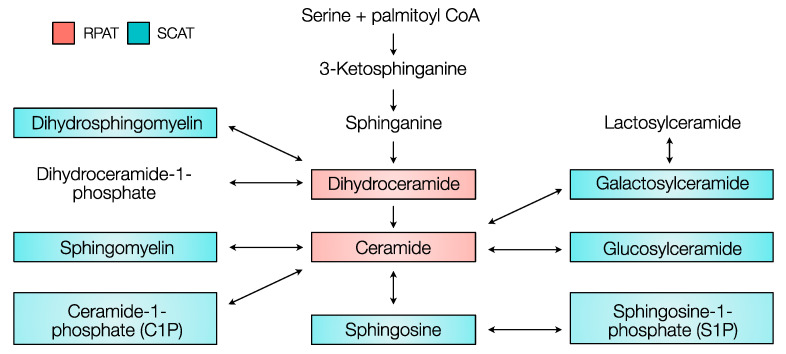
The concentration of sphingolipids across the sphingolipid pathways in subcutaneous (SCAT) and retroperitoneal adipose tissue (RPAT). Dihydroceramide and ceramide were more concentrated in RPAT (red) than in SCAT. Dyhydrosphingomyelin, sphingomyelin, ceramide-1-phosphate, sphingosine, sphingosine-1-phosphate, glucosylceramide, and galactosylceramide were more concentrated in SCAT (blue) than RPAT. There was no statistical difference in 3-ketosphinganine, sphinganine, dihydroceramide-1-phosphate, and lactosylceramide between RPAT and SCAT. The sphingolipid pathway map was drawn according to Merrill [8] and Maceyka and Spiegel [9].

Connecting all sphingolipids with the sphingolipid metabolic map, a unique distribution pattern is shown in Figure 7. Our results showed that the sphingolipid species that act downstream of 3-ketosphinganine, such as DHCers and Cers, were more concentrated in RPAT (red in Figure 7), while sphingolipids that act downstream of DHCers and Cers, such as DHSMs, Sphs, S1Ps, SMs, and glycosphingolipids, were more concentrated in SCAT (blue in Figure 7). This pattern suggested that there was a ceramide accrual in the de novo synthesis pathway in RPAT due to a higher influx rate of substrates at the origin of the de novo synthesis, due to a lower transformation rate of ceramide at the end of the synthesis, or due to a combination of both [1].

The higher substrate influx rate in RPAT could be supported by the greater dynamics of adipose mass and higher HSL activity in bovine RPAT [19]. It was shown that the adipose mass of RPAT in periparturient German Holstein cows had a greater fluctuation than that of SCAT [15,20]. Additionally, RPAT had a higher HSL phosphorylation at residues 563 and 660, as detected by Western blot analysis, indicating greater enzyme activation [13]. A similar experiment performed in rodents also showed a higher HSL phosphorylation at residues 563 and 660 in the visceral adipose tissue under forskolin stimulation, compared with the subcutaneous adipose tissue [21]. Together with the greater adipose mass in abdominal adipose depot (66.7% of total body fat) than the subcutaneous adipose tissue (17.9% of total body fat) [22], it is suggested that triglycerides stored in RPAT undergo greater facilitated hydrolysis into non-esterified fatty acids (NEFA) than SCAT. Meanwhile, palmitic acid is one of the most prevalent fatty acids among circulating NEFA in cattle [23]. The influx of palmitic acid could drain into the de novo synthesis pathway and result in a higher concentration of ceramide [24,25].

In addition to the high influx rate at the origin of the de novo synthesis pathway, the inhibition in the transformation or a backward synthesis of ceramide should also be considered as a possible source of the accrual of ceramides [1]. In the sphingomyelinase pathway, acid sphingomyelinase (ASMase) is the enzyme converting sphingomyelin into ceramide, under the activation of oxidative stress, pathogens, and the proinflammatory cytokine interleukin-1β (IL-1β) [26]. In dairy cows, Ji et al. demonstrated that the IL-1β mRNA signal was higher in the mesenteric adipose tissue than the subcutaneous depot [12]. Hence, the higher concentration of ceramide in the RPAT profile could be the consequence of the increased enzyme activity of sphingomyelinase, driven by proinflammatory signals. The enzyme that acts in the opposite direction, sphingomyelin synthase (SMS), converts ceramide to sphingolipid in the endoplasmic reticulum [27]. Although the activation mechanism and the physiological role of SMS in adipose tissue in dairy cattle has not yet been identified, it is evident that SMS could downregulate the reactive oxidative species (ROS) level by catalyzing the conversion of ceramide into sphingomyelin [28], opposing the action of ASMase. Further mechanistic studies are warranted to elucidate the role of these metabolic pathways in driving adipose depot specific sphingolipid distribution.

Comparing two sources of ceramide accrual, Rico et al. demonstrated that the de novo synthesis pathway might play a more crucial role than the sphingomyelinase pathway in dairy cattle physiology [29]. It was shown that cows with an intravenous triglyceride (TAG) infusion had a higher ceramide synthase 2 (CerS2) mRNA expression, compared with the control. The high level of CerS2 indicated an upregulation of the de novo synthesis pathway as CerS2 is the enzyme promoting the synthesis of C22:0- and C24:0 ceramide [30]. Additionally, it was shown that the SM concentration was not altered with the TAG level, indicating that the sphingomyelinase pathway was not involved in the surge of ceramide. Thus, this provided compelling evidence that the de novo synthesis pathway was more important than the sphingomyelinase pathway in contributing to the ceramide accrual [2]. The salvage pathway could, but to a lesser extent, contribute to the higher concentration of ceramide.

### 3.2. Insulin Resistance, Inflammation, and Oxidative Stress in RPAT and SCAT

Our data showed that ceramides were more concentrated in RPAT and less concentrated in SCAT. As ceramides are the upstream regulator of Akt, confirmed in an ex vivo study in Holstein steers [31], this ceramide distribution suggested that RPAT would be more associated with insulin resistance because of its ceramide profile. Ceramide has been suggested to be a mediator of obesity and insulin resistance. Previously, it was shown that ceramide could bind with PP2A inhibitor 2 (SET), releasing its inhibitory function to protein phosphatase 2 (PP2A) for the inactivation of Akt [32]. Recently, it was shown that ceramide could inhibit Akt by dephosphorylating Ser 473 [33], which was in line with bovine adipocytes research using C2:0-ceramide [31]. Furthermore, it was demonstrated that dairy cows with ceramide accrual in plasma, liver, and skeletal muscle had higher lipolytic activity and lower insulin sensitivity [3]. This provides evidence that adipose tissue with more ceramides would more suppress insulin sensitivity and have an enhanced sphingolipid dynamic. In contrast to SCAT, Kenéz et al. demonstrated that Akt and HSL phosphorylation were greater in the insulin signaling pathway in dairy cow RPAT during the peripartum period [11]. This indicated that RPAT may be more sensitive and responsive in insulin signaling than SCAT. As RPAT had a greater concentration of ceramides, RPAT may likely have a sphingolipid profile more associated with insulin resistance. Hence, RPAT may contribute to the total insulin sensitivity greater in dairy cattle.

Both insulin sensitivity and the inflammatory response were found to be different between RPAT and SCAT in cows [12]. Here, we observed that ceramides were more concentrated in RPAT, whereas sphingosines and S1Ps were more concentrated in SCAT. The distribution of Cers, Sphs, and S1Ps in RPAT and SCAT may provide an explanation in the different expression of proinflammatory cytokines in the two adipose depots. Ceramide, derived from the transformation of sphingomyelin, was shown to affect the proinflammatory cytokine IL-1β and tumor necrosis factor (TNF) signaling pathway [34]. Down to the ceramide species level, Brodlie et al. showed that C16:0, C18:0, and C20:0-ceramide levels were increased in the lower airway epithelium in human patients with lung inflammation [35]. Furthermore, sphingomyelinase, the enzyme transforming SM to Cer, was shown to be expressed to a greater extent in inflamed adipose tissues than the noninflamed adipose tissues in humans [6]. Collectively, these studies showed a strong correlation between ceramides and inflammation via the sphingomyelinase pathway. In addition to ceramides, Samad et al. demonstrated that Sphs and S1Ps could also increase the mRNA expression of proinflammatory proteins (TNF-α, monocyte chemoattractant protein-1 (MCP-1), IL-6, and keratinocyte-derived chemokine (KC)) [19]. In particular, Sphs induced more TNF-α expression, and S1P induced more IL-6 mRNA expression in 3T3-L1 adipocyte cell culture, compared with C2:0- and C6:0-ceramide. These studies showed that not only ceramide, but also its downstream sphingolipid species, such as Sphs and S1Ps, could be associated with inflammation. In dairy research, Ji et al. demonstrated that the proinflammatory cytokine (IL-1β, IL-6R, chemokine (C-C motif) ligand 2 (CCL2), and chemokine (C-C motif) ligand 5 (CCL5)) mRNA expression of omental and mesenteric adipose tissue was greater than the subcutaneous adipose tissue in overfed dairy cows [12]. As RPAT had a greater proinflammatory cytokine signal and a greater concentration of ceramides, it is suggested that RPAT is associated with a stronger inflammatory response, compared with SCAT.

In addition to proinflammatory signaling, it was shown that ceramide and sphingosine could also be the downstream mediators of oxidative stress, and regulate the apoptosis signaling pathway in both human and rat cell lines [36]. Here, we observed that ceramides were more concentrated in RPAT, whereas sphingosines were more concentrated in SCAT. These are two important mediators in regulating apoptosis under oxidative stress. Goldkorn et al. demonstrated that ROS such as hydrogen peroxide (H_2_O_2_) could activate sphingomyelinases and promote the transformation of SM to Cer in the tracheobronchial epithelial cells [37]. The surge of ceramide from the transformation could, therefore, activate cathepsin D and proapoptotic protein, BH3 interacting-domain death agonist (BID), to induce apoptosis [1,38]. Additionally, the elevated ceramide could be converted into sphingosine, acting as a second messenger to induce apoptosis by inhibiting mitogen-activated protein kinase (MAPK) activity [39]. Specifically, Osawa et al. demonstrated that C16:0-ceramide induced apoptosis in rat primary hepatocytes [40], and Seumois et al. showed that C16:0 and C24:0-ceramide are proapoptotic signals in human blood neutrophil cells [41]. These findings showed that ceramide and sphingosine are important mediators in response to oxidative stress and, therefore, induce apoptosis. Although the oxidative stress level of two adipose tissues in dairy cattle was not measured, it was shown that the visceral depots in mice are more sensitive to oxidative stress than the subcutaneous depots by comparing the stress c-Jun N-terminal kinase (JNK) and MAPK signaling pathway [42]. Thus, the visceral adipocytes might be more susceptible to apoptosis than the subcutaneous adipocytes, in agreement with a human preadipocyte study [43]. As ceramide is the precursor of sphingosine, ceramide might take a more important role in apoptosis signaling. Therefore, it is suggested that the sphingolipid profile of RPAT would be more associated with oxidative stress and apoptosis, compared with SCAT.

To conclude, this study revealed that the sphingolipid profiles differed between bovine RPAT and SCAT; in particular, the concentration of ceramides was higher in RPAT than in SCAT. This suggested that the activity of the pathways of sphingolipid metabolism, such as the de novo synthesis of ceramide, was also different in RPAT and SCAT. Consistent with previous findings, this indicated that the physiological role of RPAT could be more responsive than SCAT in insulin signaling, proinflammatory signaling, and oxidative stress response. More research has to be done to understand the metabolic stimuli and signaling pathways of other sphingolipids, such as DHCer, Sph, S1P, and C1P in adipose tissue, to provide a comprehensive comparison of the physiological role of RPAT and SCAT.

## 4. Materials and Methods

### 4.1. Animals and Sampling

Six German Holstein bulls, intended for beef production, were used for adipose tissue sample collection for this study. Animals were kept at the Educational and Research Center for Animal Husbandry, Hofgut Neumuehle (Muenchweiler a.d. Alsenz, Germany). They were a subgroup of a larger cohort used for a nutritional trial, approved by the relevant Department for Animal Welfare Affairs (Landesuntersuchungsamt Rheinland-Pfalz, Koblenz, Germany) in agreement with the German Animal Welfare Act (permit number: G-17-20-070). Bulls were housed on a slatted floor with rubber mats in groups of four animals and were fed a total mixed ration based on grass silage and corn silage. Although the current study’s objectives did not include the study of nutritional influence on adipose depot dependent sphingolipid abundance, the diet of three of the six bulls included an additional 6 kg per day concentrate for 7 months before slaughter. This is because the larger nutritional trial that these animals derived from had a focus on intensive and moderate fattening regimens (intensive: 11.4 vs. moderate: 10.2 MJ metabolizable energy (ME)/kg of dry matter (DM)). However, the number of replicates per dietary group (*n* = 3) was considered to be insufficient in terms of statistical power to confirm or reject any dietary effects and, thus, this aspect was not further investigated. Bulls were slaughtered at an age of 20 months (live weight 755 ± 73 kg; means ± SD), and tissue samples of the subcutaneous adipose depot (at the tail head) and the retroperitoneal adipose depot were collected within 30 min. Samples were immediately rinsed in ice-cold physiological saline solution and cut into approximately 100 mg pieces, before snap-freezing them in liquid nitrogen. Samples were then stored at −80 °C until analysis.

### 4.2. Sphingolipid Measurement

Samples were submitted to The Metabolomics Innovation Center (TMIC UVic Node at The University of Victoria, Genome BC Proteomics Centre, Victoria, BC, Canada) for sphingolipid profiling. The detailed experimental procedures were described in previous studies [44]. In brief, lipid extraction was accomplished by methanol–chloroform bilayer separation. After the samples were mechanically homogenized with metal beads, 10 mL per mg of methanol–chloroform (5:2, *v/v*) was added into the mixture with 0.1 mg/mL antioxidant butylated hydroxytoluene (BHT). The mixture was then homogenized, sonicated, and centrifuged in an Eppendorf 5420R centrifuge (Eppendorf AG, Hamburg, Germany) for 15 min at 21,000× *g* and 10 °C for the first lipid extraction. The precipitated pellet was homogenized with methanol–chloroform (1:1, *v/v*) at 10 µL/mg tissue weight again using the same set-up for the second lipid extraction. After centrifugation, the clear supernatant was pulled and dried by nitrogen gas at 30 °C. The residue was dissolved in methanol with two internal standards: C17:0-sphinganine as the positive-ion internal standard and C17:1-sphingosine-1-phosphate as the negative-ion internal standard.

After the lipid extraction, the mixed standard stock solution (S1) was serially diluted with methanol in a ratio of 1:4 (*v/v*) into stock solution S2 to S10. Then, 10 μL of sample was injected into UPLC–MS/MS for lipid detection. The Waters UPLC system (Milford, MA, USA) coupled with a 4000 QTRAP mass spectrometer (AB Sciex, Framingham, MA, USA) was operated in the multiple reaction monitoring (MRM) mode. The mobile phase in UPLC was made of 0.01% formic acid in water and acetonitrile-isopropanol (2:1, *v/v*) for binary-solvent gradient elution. The UPLC–MS/MS in MRM mode data were documented with Sciex Analyst software and processed with Sciex MultiQuant software (AB Sciex). The concentrations of sphingolipids were quantified from the calibration standard curves with the measured peak areas. A total of 77 sphingolipid species were targeted and 70 species were detected, including 24 species of ceramide (Cer) and dihydroceramide (DHCer), 16 species of sphingomyelins (SM), two species of dihydrosphingomyelin (DHSM), 11 species of ceramide-1-phosphate (C1P) and sphingosine-1-phosphate (S1P), nine species of galactosylceramide (GalCer) and glucosylceramide (GluCer), lactosylceramide (LacCer), and eight species of 3-ketosphinganine, sphinganine (DHSph), and sphingosine (Sph). The raw data of sphingolipid concentrations are provided in the Supplementary Materials (Table S1), with the mean and SD for each sphingolipid species.

### 4.3. Statistical Analyses

To visualize the fold-change and *p*-value of a *t*-test analyzed between SCAT and RPAT, a volcano plot was drawn in R version 4.0.0 with the package ggplot2. The thresholds for log_2_(fold-change) were set at −0.5 and 0.5, and the threshold for *p*-value was set at 0.05. To show the correlation of tissue samples and sphingolipids, a principal component analysis (PCA) biplot was drawn in MetaboAnalyst 4.0 (https://www.metaboanalyst.ca) [45]. Data were log-transformed and Pareto-scaled. To visualize the individual variation between animals, a heatmap with dendrograms was drawn in MetaboAnalyst 4.0. Data were log-transformed and Pareto-scaled. Samples and features were clustered in Euclidean distance with the Ward algorithm. To show the distribution of sphingolipids in SCAT and RPAT, bar plots of the three pathways of interest were drawn in GraphPad Prism version 8.3.0 for Mac (GraphPad Software, La Jolla, CA, USA). The *p*-values between SCAT and RPAT were calculated by paired Student’s *t*-test with false discovery rate (FDR) correction in Benjamini–Hochberg procedure, using the function P.adjust in the R package stats. The standard deviation (SD) was shown by the error bar. Levels of statistical trends and statistical significance were indicated as follows: * *p* < 0.05; ** *p* < 0.01.

## Supplementary Materials

The following figure and table are available online at https://www.mdpi.com/2218-1989/10/11/473/s1: Figure S1. Principal component analysis (PCA) biplot visualizing the relationship of tissue samples (black) and sphingolipids (red); Table S1. The sphingolipid concentrations (raw data) of bovine SCAT and RPAT.
