# Supplementary material for: Sphingolipid Profiling Reveals Different Extent of Ceramide Accumulation in Bovine Retroperitoneal and Subcutaneous Adipose Tissues
Source: Metabolites. 2020 Nov 19;10(11):473. doi: 10.3390/metabo10110473 (PMC7699355; doi:10.3390/metabo10110473)
Supplement: Supplementary file 1 [file metabolites-10-00473-s001.zip › supplementary materials-final/Figure S1.docx]

Supplementary Materials: Sphingolipid Profiling Reveals Different Extent of Ceramide Accumulation in Bovine Retroperitoneal and Subcutaneous Adipose Tissues

Yue Hei Leung ^1^, Sonja Christiane Bäßler ^2^, Christian Koch ^3^, Theresa Scheu ^3^, Ulrich Meyer ^4^,
Sven Dänicke ^4^, Korinna Huber ^2^ and Ákos Kenéz ^1,^*

^1^ Department of Infectious Diseases and Public Health, City University of Hong Kong, Hong Kong; yuehleung4-c@my.cityu.edu.hk

^2^ Institute of Animal Science, University of Hohenheim, 70599 Stuttgart, Germany; Sonja_10394@gmx.de (S.C.B.); Korinna.Huber@uni-hohenheim.de (K.H.)

^3^ Educational and Research Centre for Animal Husbandry, Hofgut Neumuehle, 67728 Muenchweiler a.d. Alsenz, Germany; c.koch@neumuehle.bv-pfalz.de (C.K.); T.Scheu@neumuehle.BV-Pfalz.de (T.S.)

^4^ Institute of Animal Nutrition, Friedrich-Loeffler-Institute (FLI), Federal Research Institute for Animal Health, 38116 Braunschweig, Germany; ulrich.meyer@fli.de (U.M.); sven.daenicke@fli.de (S.D.)

***** Correspondence: akos.kenez@cityu.edu.hk


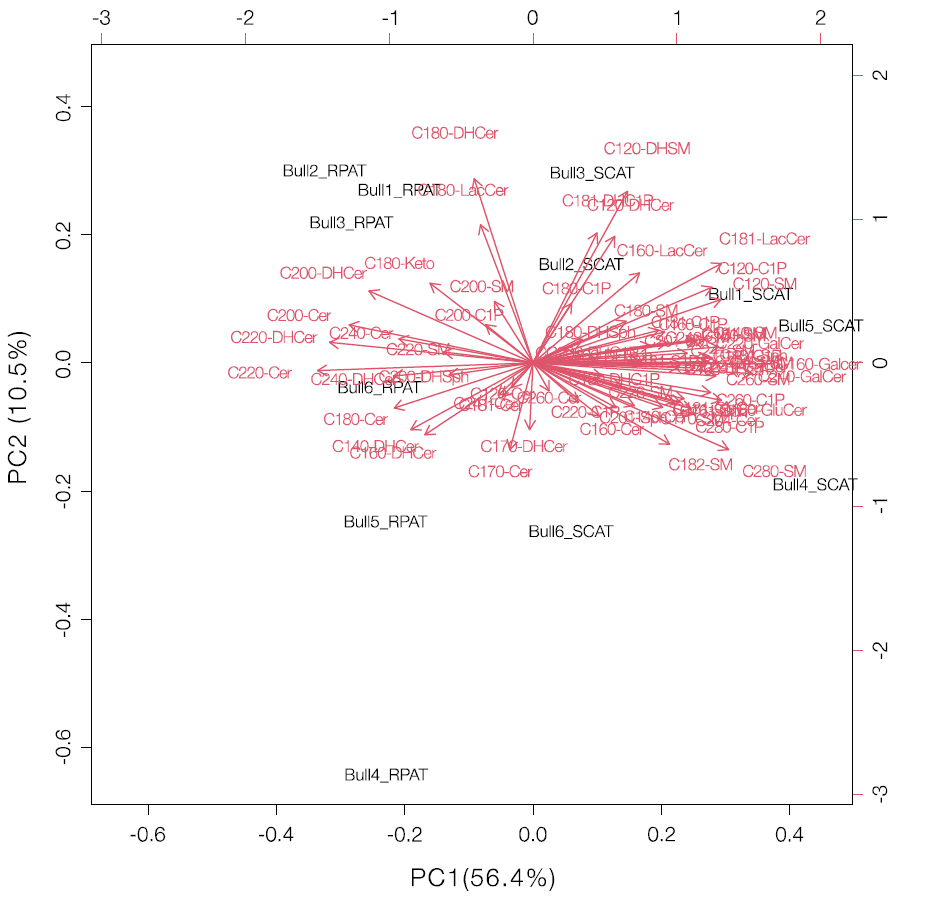


**Figure 1.** Principal component analysis (PCA) biplot visualizing the relationship of tissue. samples (black) and sphingolipids (red). Data were log-transformed and Pareto-scaled. In the PCA scores plots, the RPAT samples were clustered on the left (PC1 < 0), and the SCAT samples were clustered on the right (PC1 > 0). In the loadings plot, sphingolipids belonging to the sphingomyelinase pathway and salvage pathway were mostly positively correlated to PC1, except for C22:0-SM and C20:0-SM; and sphingolipids belonging to the *de novo* synthesis pathway were mostly negatively correlated to PC1.
